# Selective Addressing of Versatile Nanodiamonds *via* Physically-Enabled Classifier in Complex Biosystems

**DOI:** 10.1021/acs.nanolett.4c06567

**Published:** 2025-03-14

**Authors:** Yayin Tan, Xiaolu Wang, Feng Xu, Xinhao Hu, Yuan Lin, Bo Gao, Zhiqin Chu

**Affiliations:** † Department of Electrical and Electronic Engineering, the University of Hong Kong, Pok Fu Lam, Hong Kong, China; ‡ School of Biomedical Sciences, Faculty of Medicine, the Chinese University of Hong Kong, Shatin, Hong Kong, China; ◧ Centre for Translational Stem Cell Biology, Tai Po, Hong Kong, China

**Keywords:** Fluorescent Nanodiamonds, NV Centers, Optically-Detected Magnetic Resonance, Selective Addressing, Fluorescent Imaging, Bioimaging, Physically-Enabled Classifier

## Abstract

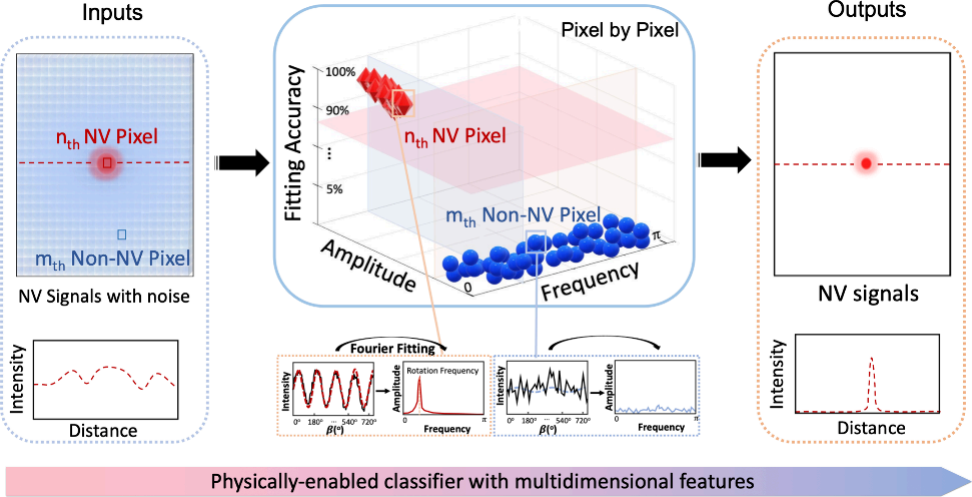

Nitrogen-vacancy (NV) centers show great potential for nanoscale biosensing and bioimaging. Nevertheless, their envisioned bioapplications suffer from intrinsic background noise due to unavoidable light scattering and autofluorescence in cells and tissues. Herein, we develop a unique all-optical modulated imaging method via a physically enabled classifier, for on-demand and direct access to NV fluorescence at pixel resolution while effectively filtering out background noise. Specifically, NV fluorescence can be modulated optically to exhibit sinusoid-like variations, providing a basis for classification. We validate our method in various complex biological scenarios with fluorescence interference, ranging from cells to organisms. Notably, our classification-based approach achieves an enhancement of signal-to-background ratio from 1.92 to 60.39 dB for fluorescent nanodiamonds in neural protein imaging. We also demonstrate a 4-fold contrast improvement in optically detected magnetic resonance measurements inside stained cells. Our technique offers a generic, explainable, and robust solution, applicable for realistic high-fidelity imaging and sensing in challenging noise-laden scenarios.

To comprehend and regulate intricate biological processes at the molecular scale, it requires spotlighted analysis on the interactions and distribution dynamics of biomolecules, such as proteins and DNA, within complex biological environments such as living cells or intact tissues.^1^ Significant efforts have targeted developing new fluorescent probes^2,3^ and imaging techniques, for applications like labeling cellular components and tracking dynamic biological processes.^4−8^ However, in practice, bioimaging is often hindered by undesirable autofluorescence, tissue light scattering, and other cluttered signaling from the spatiotemporal dynamics of cellular components.^9−11^ Moreover, fluorescent probes generally encounter issues in color fading and degradation over time, limiting their durability and photostability for long-term observation.^12^ To address these challenges, it is essential to develop robust fluorescent probes and imaging techniques that can directly access the fluorescence signals of targeted agents, particularly amidst complex and varying backgrounds such as living cells or tissues.

Among the numerous fluorescent probes, fluorescent nanodiamonds (FNDs) with nitrogen-vacancy (NV) centers stand out as revolutionary agents due to their remarkable biocompatibility,^13^ unlimited photostability, chemical robustness, and nanoscale quantum sensing capability at room temperature.^14,15^ Those outstanding superiorities, together with the biologically favorable near-infrared emission wavelength,^16^ promote FNDs as a versatile tool in cellular labeling, tracking, and quantum sensing of nanoscale physical quantities such as temperature, magnetic fields, and rotations.^17−20^ Nonetheless, it remains challenging to detect a single FND signal contaminated by complex bioenvironments due to its relatively weak signal energy and easy crosstalk.^21^ To improve FND detection efficiency, researchers have made efforts in developing NV-based background-free imaging techniques. The developed techniques (as summarized in Table S1,^22−30^Supporting Information) can be broadly categorized into three types of approaches for modulating the spin-dependent fluorescent intensity of NV centers: magnetic-,^23,25,28,29^ microwave-,^24,28,30^ and optically modulated^22,26,27^ approaches. However, despite those achievements to date, each type has its own limitations. For instance, magnetic approaches are sensitive to environmental magnetic noise, which degrades image quality. Microwave-modulated imaging may cause thermal damage to fragile biological samples,^22^ narrowing its scope of applications. While for the optical modulation,^22^ the typically achieved contrast of FNDs was around 3%, insufficient for high-fidelity imaging applications.

Recently, we have developed an optical modulation approach to track multidimensional movements of FNDs for cell–matrix interactions.^31^ The spectrum of FNDs showed a sinusoid-like pattern with high modulation contrast of 20–68%.^32^ These physical properties provide a basis for resolving FND detection efficiency. Here, we propose a unique codesign of an all-optical polarization-modulated system and a physically enabled, robust, and explainable classifier to achieve selective addressing of FNDs at single pixel-scale resolution. Utilizing the multidimensional features of extracted NV fluorescence signal as detection criterion, this classifier robustly distinguishes NV pixel signals from non-NV pixel signals. We demonstrated our method by observing FNDs within cultured stained cells, inside zebrafish embryos with autofluorescence, and by utilizing FNDs as immunofluorescent labels in neural tissue of mouse brains. Notably, we enhanced the signal-to-background ratio (SBR) of FND imaging from 1.92 to 60.39 dB in neural protein imaging and the optically detected magnetic resonance (ODMR) measurements of FNDs in cultured stained cells up to 4-fold, despite the presence of disturbing complex environmental fluorescence. Thus, we validated the effectiveness and adaptivity of our method in multiple biological scenarios, showcasing its superior performance in bioimaging. Compatible with existing quantum sensing schemes, our method demonstrates great potential in challenging noise-laden laboratory applications, such as high-resolution imaging and sensing in tissues, live cells, and small animals.

To develop a broadly applicable technique for selective addressing of FNDs, we constructed a self-built wide-field system (Figure S2A) based on the optical polarization dependence of NV centers. An NV center is a lattice defect within nanodiamonds (Figure S2B), comprising a substitutional nitrogen atom and adjacent vacancy.^14^ At room temperature, NV centers emit strong red fluorescence (640–780 nm) under 532 nm green optical excitation (Figure S2B), which can be collected by an EMCCD camera.^15^ Here, we inserted an electrically rotating half-wave plate (HWP) in the excitation path to modulate laser directions (Figure S2A). NV fluorescence emission varies with the linear polarized laser that forms different angles to the NV axis.^31^ Theoretically, the optical modulation follows the polarization-selective excitation rule of a single NV center:^31,33^
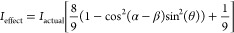
1where *I*_effect_ is the effective excitation laser power, *I*_actual_ is the actual excitation laser power; θ and α are constants related to NV axis projection angles. The definitions of θ, β, and α are shown in Figure 1A and in the Supporting Information. The red dashed curve presents the sinusoidal NV signal modulated by the HWP, and the bottom blue dashed curve indicates the background non-NV signal with random fluctuations. A detailed description can be found in Experimental Methods in the Supporting Information.

**Figure 1:**
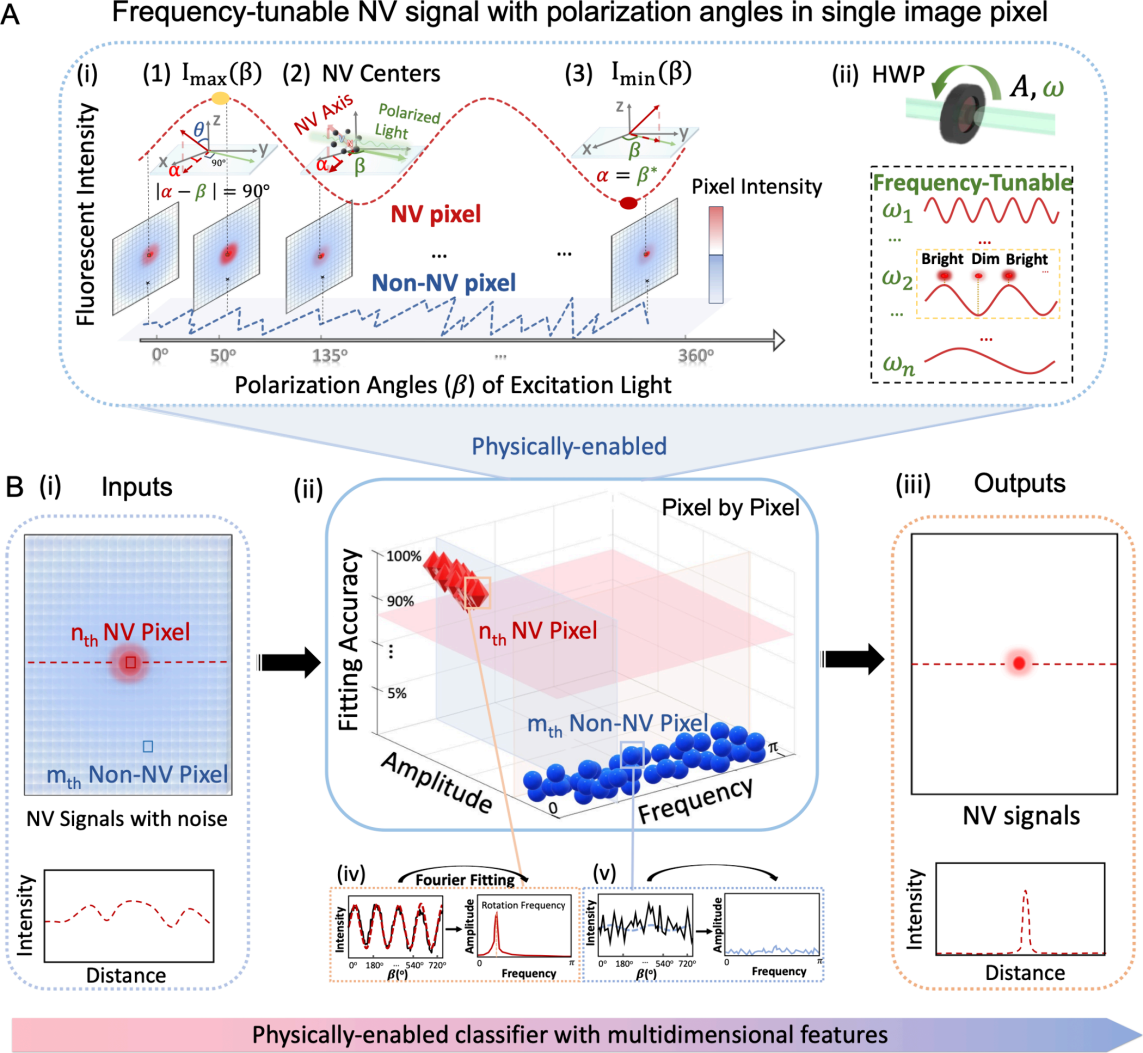
Principle and design of the physically enabled classifier with simple optical modulation for the selective addressing of NV pixels. (A) (i) Schematic illustration showing the optically modulated NV pixel signal in the obtained image, together with the non-NV pixel signal. (1–3) The NV axis orientation (light red solid arrow) with corresponding projection (dark red dashed arrow, α) to the sample plane and laser polarization direction (light green arrow, β) are illustrated, where the sample plane serves as reference.^31^ Details of the defined angle changed by HWP can be found in the Supporting Information. (ii) The HWP rotation (upper illustration) controls the intensity amplitude A and the angular frequency ω (ω = 2*πf*) of NV fluorescence signals. (B) The designed automatic NV imaging machinery and processing pipeline: the 2D images of interest with equidistant polarization angles are encoded as a 3D tensor, serving as the inputs. (ii) The physically enabled classifier with multidimensional features to automatically distinguish NV targets from background noise (non-NV) combining Fourier fitting and analysis. (iii) The high-contrast, robust, and selective NV imaging outputs against system noise in this encoder–decoder framework.

The modulated NV curve extracted from a series of image pixels shows a periodic property of a single-frequency and sinusoidal-like signal. After sufficient and proper optical modulation, this polarization-dependent optical property of NV centers exhibits high contrast ranging from 20% to 68% in fluorescence intensity.^31^ The FND contrast is defined as
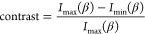
2

In addition, the HWP can rotate at different speeds, providing a flexible and tunable way for controlling the frequency of modulated NV signal accordingly (Figure 1A(ii)). Therefore, this regular and optical modulation can be utilized to provide a physical basis for achieving the selective addressing of NV centers.

After completing optical hardware modulation, we then devised a physically enabled classifier utilizing multidimensional features by algorithms as NV imaging machinery to selectively address NV fluorescence and filter out background fluorescence. Mathematically, we algebraically encoded those 2D images of interest obtained at equidistant excitation polarization angles as a 3D tensor (tensor size: *H* × *W* × *K*; *H* and *W*: 2D image size; *K*: number of polarization angles), serving as inputs to the designed imaging framework (Figure 1B). Each pixel at the same position across images was extracted together to form as a one-dimensional signal, a sequence varying with laser polarization angles. The NV pixel signal manifests as a periodic modulation property that could be well characterized as a single sine-wave model. Background pixel signals cannot be regularly modulated optically and exhibited random low-intensity fluctuations, modeled as white noise.

The quantitative multidimensional features of tensor data are extracted as fitted frequency, amplitude, and fitting accuracy, through advanced Fourier analysis. (Details can be found in Experimental Methods in the Supporting Information.) Compared to non-NV pixels, the targeted NV pixel signals exhibit much larger modulated amplitude, fixed fitted frequency, and significantly higher fitting accuracy. Specifically, the signals of sparse NV-pixels (around or less than hundreds), which are excited by polarized laser, adhere to a sinusoidal model for which its frequency is uniquely determined by experimental settings. Here, the frequency can be tuned via changing the HWP speeds, offering flexibility for our imaging and sensing scheme. The wide-range frequency modulation of NV signals varies from the Hz to kHz scale, as shown in Figure S3. Yet for the massive non-NV pixels (million scale), they abide by a white noise distribution and have a uniform power spectral density, leading to random small amplitudes. As demonstrated in various experimental scenarios, the fitting accuracy of the NV pixel achieves a sinusoidal fitting level of more than 80% compared to that of non-NV pixels, which is below 15%. These mathematical characteristics provide criterion for pixel-level classification, allowing for NV selective addressing in practical images. By integrating these features, all pixels in the image can be robustly classified into NV pixels and non-NV pixels.

Hence, a multidimensional classifier is designed accordingly as a decoder to classify NV targets automatically and robustly from background noise (non-NV pixels), implemented via an efficient algorithm. Algorithmically, NV-selective images can be generated as outputs (Figure 1B, right) with significantly enhanced SBR, which we have also validated across various data modalities and biological scenarios. In experimental verifications, the designed classifier is robust and resilient to common data distortion like frame deviation and being out-of-focus, which frequently occur in lab setups. This encoder–decoder framework enables high-contrast, robust, and easy extraction for the selective addressing of the NV signal against system noise and calibration error.

To demonstrate the effectiveness and universality of our method against various noise sources typically encountered in bioimaging, we validated it in four fluorescent imaging scenarios (Figure 2A–H). These scenarios included static background noise from LED light and dynamic background noise from fluorescent dyes, cells stained with red fluorescence dye, and zebrafish embryos.

**Figure 2:**
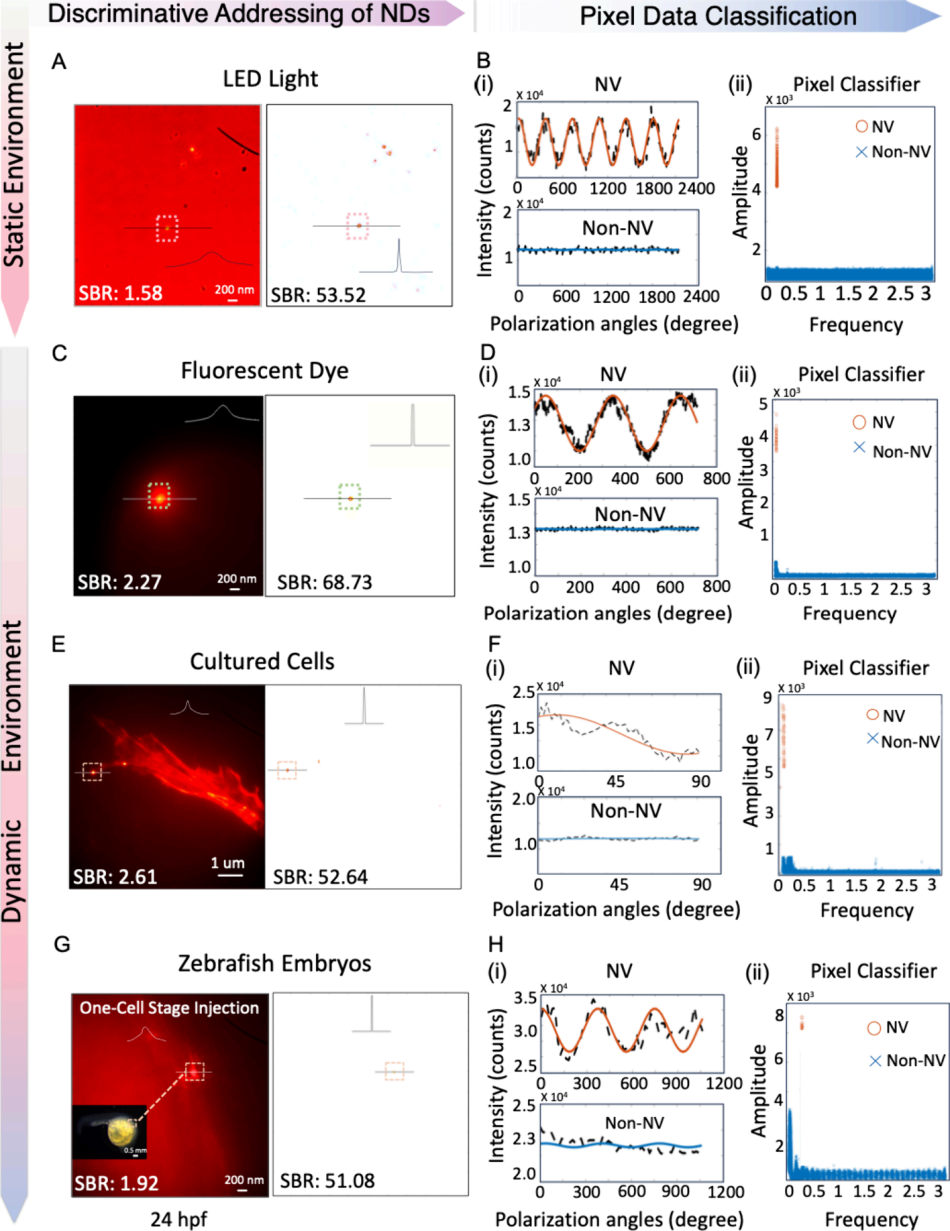
Selective addressing of NV targets via the physically enabled classifier, in static and dynamic environments. (A, C, E, G) The left panels are original fluorescent images of FNDs exposed to the noise environment of LED Light, fluorescent dye, cultured 3T3 cells stained with Alexa Fluor 647 Phalloidin, and zebrafish embryo with autofluorescence, respectively. The 2D images were captured under equidistant polarization angles. The right panels are selective imaging of FNDs by the classifier, with all the background noise filtered out. (B, D, F, H) (i) The NV and non-NV original signals and their fitting in comparison. (ii) The pixel classifier for NV and non-NV pixel classifications.

In a proof-of-concept study, FNDs were irradiated by LED light (275–950 nm) with a constant intensity (Figure 2A). The fluorescence intensity of FNDs was comparable to or lower than that of the LED light. Our classifier successfully distinguished those luminous FNDs while significantly reducing the blurring effect from point spread function (PSF) and effectively filtering out unwanted background noise. The fluorescence intensity variations across the black-lined FND (Figure 2A) showed a notable decrease in the full width at half-maximum (FWHM) of the FND intensity curve. The classification results and extracted features, including the NV and non-NV raw pixel data, frequency, and amplitude via Fourier fitting, are presented (Figure 2B,D,F,H and Figure S4). In Figure 2B, the fitted amplitude of NV is around 12000. The fitted frequency is around 0.21, matching the experimental setting with fitting accuracy over 85%. Yet for the non-NV pixels, the fitted amplitude by modulation is around a hundred, and the fitted frequency is randomly spaced with fitting accuracy below 5%. By assessing these multidimensional features, NV and non-NV pixels are well-classified, enhancing the imaging quality of SBR from 1.58 to 53.52 dB.

We then exposed FNDs into drops of red fluorescent dye (emission: 650–750 nm), as a dynamic environment for FNDs. Our algorithm quantified the multidimensional features of the raw image data pixel by pixel. In Figure 2C, we obtained an NV selective image of a single FND by the customer-designed classifier, improving the SBR from 2.27 to 68.73 dB.

We further validated the bioapplicability of our scheme in live cultured cells stained with the red fluorescent dye (Figure 2E,F). FNDs were introduced into 3T3 cells by endocytosis, and cells were stained with Alexa Fluor 647 Phalloidin red (AF-Red). The fluorescence emission spectrum of AF-Red (Figure S5) overlaps with that of NV centers, hampering FND detection. Through our robust classification scheme, we obtained high-contrast and clearer selective images of FNDs within live cells. The observed bright spots were verified as NV centers through ODMR measurements (Figure S5). The SBR was notably improved from 2.61 to 52.64 dB. The acquisition time of the obtained images was 3.2 s, shorter than the time frame of various biological events like cellular metabolism.^22,34^

Finally, we demonstrated our method in zebrafish embryos with strong autofluorescence in yolk at the initial growth stage, a popular model for disease modeling and drug screening.^35^ FNDs were injected into zebrafish embryos at one-cell stage, fertilized for 24 h before imaging. The concentration of injected FNDs was controlled to avoid aggregations, allowing for observing sparsely distributed FND inside the zebrafish embryo (Figure 2G). The SBR was improved from 1.92 to 51.8 dB, demonstrating the effectiveness and applicability of our method in an in vivo system.

These comprehensive study results demonstrate our multidimensional classifier can address NV centers robustly regardless of ambient disturbances, verifying its robustness and accuracy for NV-center detection, against various noise sources in realistic bioimaging applications.

To utilize our method in quantum sensing, we then applied our scheme to ODMR measurements of FNDs inside the living cell. We designed a new protocol to capture the wide-field fluorescent images for ODMR (Figure S6). Our classifier was used in the selective imaging of FNDs and the enhancement of ODMR detection. The cultured cell was stained by Alexa Fluor 647 Phalloidin with an emission range similar to that of FNDs, for which the emittance wavelength larger than 638 nm can be acquired by the optical wide-field system as the background noise. Inside the green circles of Figure 3A are FND 1, 2, and 3, marked as the red bright spots. These bright spots were verified as FNDs by ODMR frequency spectroscopy (Figure S6). The selective imaging of FNDs inside a single stained cell was achieved by our method (Figure 3B). The FNDs inside the green circles of Figure 3B are the same as in Figure 3A. The fluorescent intensity variations of FND 1, 2, and 3 are presented in Figure 3C,D showing a successful background removal and higher resolution. Notably, the closely aggregated FND 1 and 2 were separated via the selective imaging, demonstrating the improved resolution of FNDs by our method.

**Figure 3:**
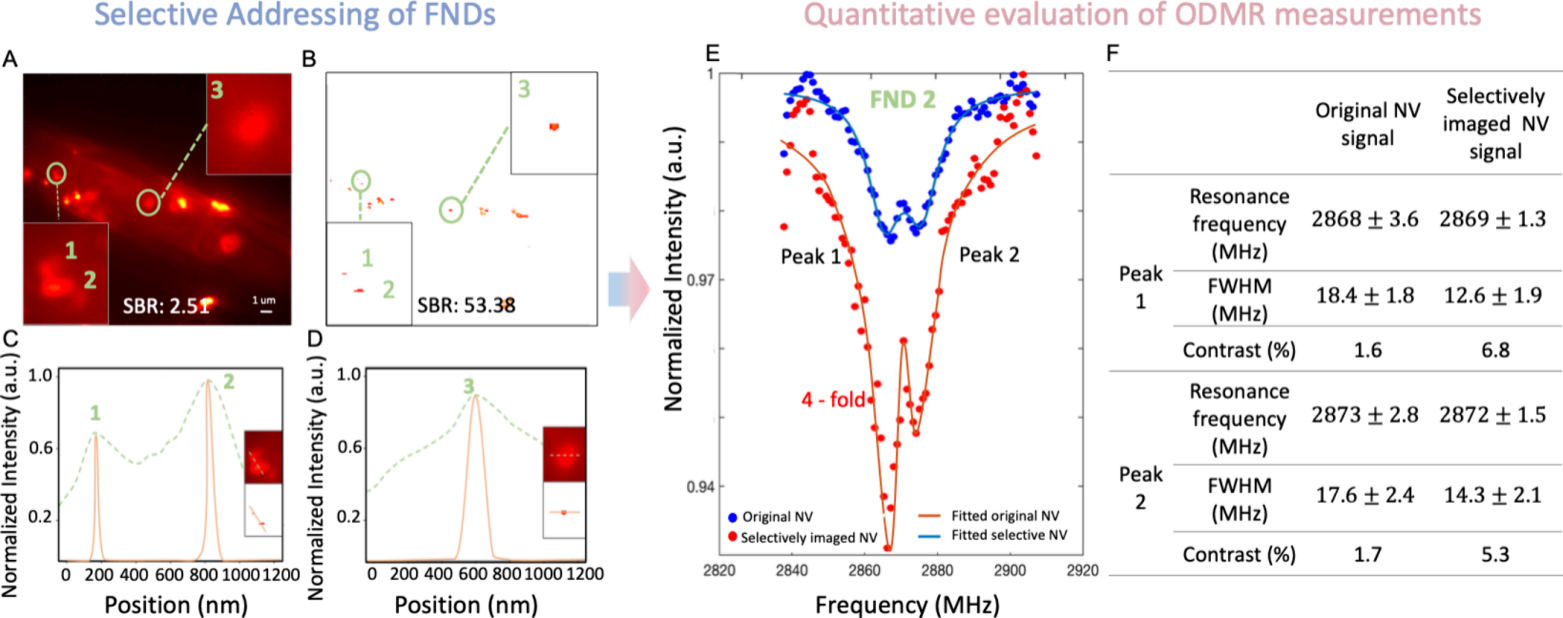
Classifier-enhanced ODMR detection of FNDs inside a cultured single cell. (A) Wide-field fluorescent images of FNDs inside single cell stained by Alexa Fluor 647 Phalloidin, captured under equidistant polarization angles. FND 1, 2, and 3 are highlighted inside the green circles, seen as the red bright dots. (B) The selective addressing of FNDs inside cells achieved by the classifier. FNDs inside the green circles are the same spots as indicated in panel A. (C and D) The fluorescent intensity of FNDs through the line across the circled FNDs at different positions. (E) ODMR curves of FND 2 in green circle of (A) with a 4-fold improved contrast by the classifier. (F) Table for concluding the FWHM and contrast of each ODMR peak in panel E. The results were represented as mean ± s.d. (from 10 independent measurements).

The illustration for the experimental ODMR detection protocol is shown in Figure S6B. In Figure 3E, the ODMR curve of FND 2 shows a 4-fold improvement on the peak contrast, with a 91% confidence interval of the Lorentz fit. The table in Figure 3F shows the performance of the FWHM and contrast of FND 2. The SBR of the original fluorescence image was 2.51 dB and was improved to 53.38 dB by our method (Figure 3B). The calculated results show that our method can enhance the ODMR measurements by filtering out those influential background noise.

We further tested our method’s applicability by observing the immunofluorescent imaging of FND conjugations as biomarkers. In Figure 4A, FNDs were functionally conjugated with secondary antibody (SAb) of IgG 488, to be applied in the immunofluorescent labeling of NeuN in mouse brain tissue (Figure S7, Supporting Information). The FND conjugations (IgG 488 @ FND) serve as the SAb to track the subcellular location of the primary antibody of anti-NeuN. Confocal images of neurons labeled with IgG 488 @ FND conjugates were shown in different fluorescent channels (Figure 4B). Under the 488 nm excitation channel, FNDs were hard to observe. Green dots in Figure 4B-ii indicate the IgG 488 fluorescence. While under the 647 nm channel, FNDs appeared as red dots, where IgG 488 cannot be observed (Figure 4B-iii). These channels independently validated the existence of FNDs. Here, we were able to visualize the subcellular distribution of FND conjugations within the nuclei regions (DAPI positive regions), where the white spots indicated the colocalization of SAb signals (IgG 488) and FNDs. The percentage of the matching white spots (Figure 4B-ii merged with b-iii) is higher than 86%, indicating the conjugation efficiency. Two control groups (middle and bottom rows of Figure 4B) confirmed the specific and successful binding of FND conjugations onto the brain tissue (Supporting Information). All these results demonstrated the specificity of FND conjugates, with successful conjugation to primary antibody Anti-NeuN.

**Figure 4:**
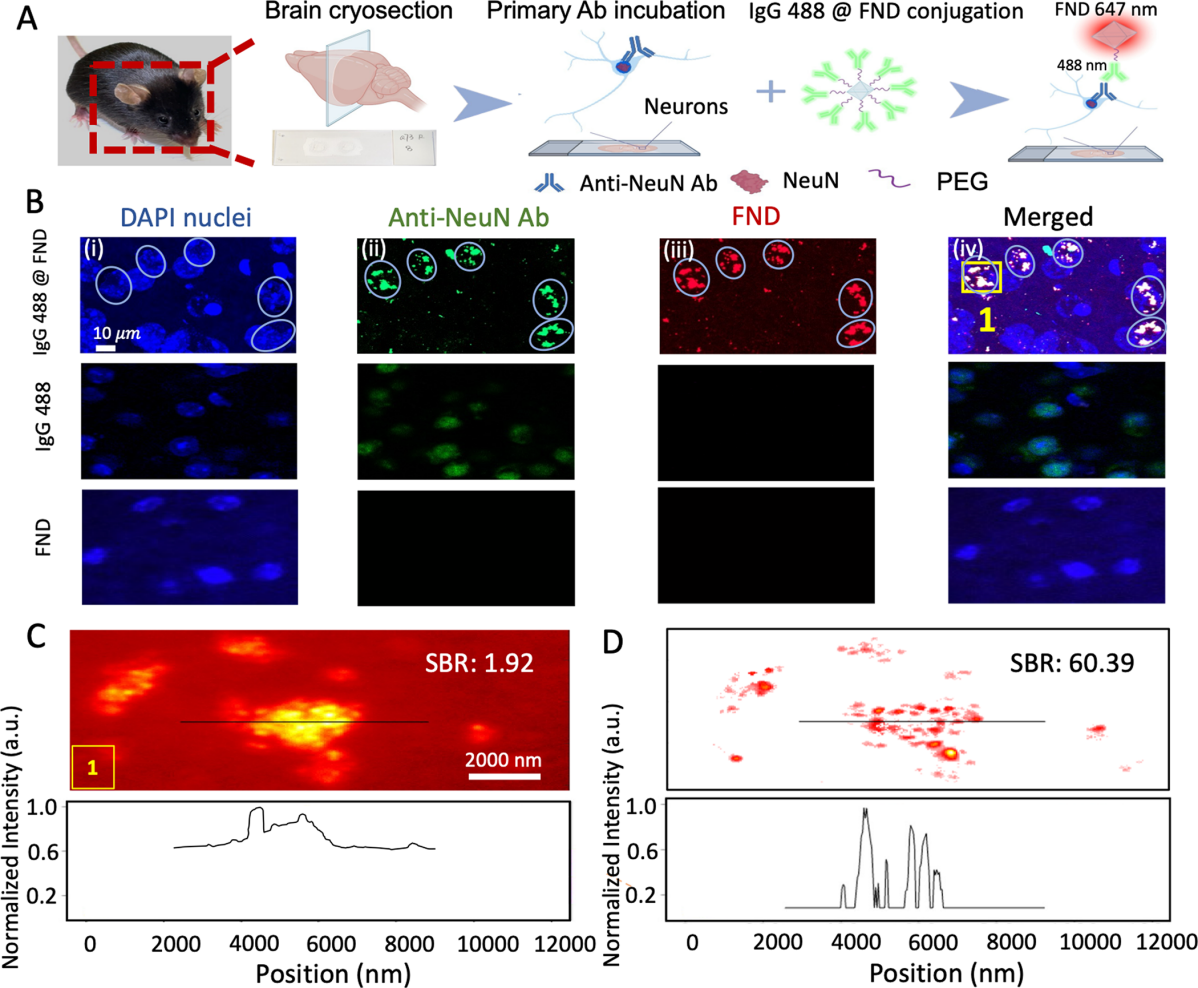
Selective immunofluorescent imaging of FNDs conjugated with SAb in brain tissue slice from an adult mouse through the classifier-based imaging method, where IgG 488 @ FND conjugates were utilized for visualizing primary antibody localization. (A) Schematic illustration showing the conjugation of FNDs with IgG 488 and its application onto the immunofluorescent labeling of the NeuN proteins in mouse brain slices. (B) First row: (i–iv) confocal images of the neuronal cells labeled with IgG 488 @ FND conjugates in different fluorescent channels (405, 488, and 647 nm). (i) Nuclei regions were visualized by DAPI (4′,6-diamidino-2-phenylindole) at 405 nm channel, shown in blue. (ii) Neuronal nucleus (NeuN) was visualized by anti-NeuN, conjugated with IgG 488 @ FND at 488 nm channel. (iii) FNDs were imaged by 647 nm channel. (iv) Merged image of i, ii, and iii visualizes the subcellular distribution of FNDs inside the nuclei, where the white spots indicate the colocalization of DAPI (i), SAb (ii), and FNDs (iii). Second and third row panels are control groups (IgG 488 and FND solution). (C) Fluorescent images of FNDs captured by the self-built wide-field system, the same location as the inset 1 with yellow box in panel iv. Bottom: immunofluorescent intensity variations across the aggregated FNDs with the white line. (D) Selective imaging of FNDs by the classifier applied in panel C, filtering out all of the background fluorescence. Bottom: the fluorescent intensity variations through the white line across the aggregated FNDs. Ab: antibody. PEG: Polyethylene glycol.

We then captured fluorescent images of brain tissue slices stained with primary antibody and FND conjugates (Figure 4C, the same location as the yellow box marked in Figure 4B), using our home-built wide-field microscope. In Figure 4C, the distribution of FND conjugates was clearly observed with strong fluorescence background due to the various fluorescent dyes and material autofluorescence during the slice preparation. FND conjugates exhibited an aggregated property on nuclei due to specific binding, impacting their applications in bioimaging and biosensing. Here, our classifier-based approach allowed us to classify and separate the aggregated FNDs, enhancing the SBR from 1.92 to 60.39 dB (Figure 4C,D). This indicates that our approach successfully reduces the background noise and enhances the clarity and specificity of the immunofluorescent imaging. Fluorescent intensity variations across white-lined aggregated FNDs (lower panel of Figure 4D) show improved resolution, with narrowed fluorescent intensity peaks for each resolved FND. Therefore, our method successfully filters out background fluorescence inside brain tissue, achieving selective addressing of FNDs. In summary, our method brings practical benefits in immunofluorescent imaging, with FND conjugations acting as biomarkers and sensing agents in biological tissue analysis.

Here, our approach employs robust atomic defects in FNDs, the NV-centers with photostability and high-modulation contrast, to ensure durable and reliable imaging for detection in diverse complex biosystems. This fluorophore-free imaging strategy also overcomes bottlenecks of noisy fluorescent bioenvironmental influences and low contrast of FNDs, hence enhancing imaging and sensing quality. Compared with existing imaging techniques (Table S1), our proposed method shows its uniqueness by employing a codesign of optical hardware and algorithm. On the hardware front, we built an all-optical modulated imaging setup for convenient and fast signal readout, which provides a simple, flexible, and tunable optical platform for controlling the modulation of NV signals. On the algorithm front, we designed a robust and explainable physically enabled classifier based on physical properties of the NV sinusoid-like pattern. Distinct from a recent imaging method using single modulation with a single parameter for background subtraction, our multidimensional feature extraction strategy offers a more reliable performance for handling multiple data modalities in a variety of noisy real-world scenarios. It also offers scalability for the amounts of data and is capable of operating at a small amount of raw data as few as several frames of modulated images, saving time for measurement and processing. This provides flexibility and reduces the experimental cost.

Biological authentication results proved that our method, grounded in theoretical principles, can achieve robust NV classification at pixel-scale resolution and image enhancement with satisfactory distinguishability, despite the presence of complex background interferences. This showcased the practical benefits of our multifeature classification method for the selective addressing of FNDs, particularly in overcoming issues like sample drift, frame-to-frame variations, and observational disturbances occurred in practical experiments. Compatible with existing quantum sensing schemes, our method demonstrates its superiority in improved detective efficiency, stability under diverse scenarios, and ease of integration into various systems.

As the first step toward intelligent, explainable, and reliable imaging technology, the follow-up extensions of our method can be developed in the following aspects: (i) On the experiment side, the temporal resolution of the optical system could be possibly improved by integrating an ultrafast polarizer module, with ongoing research indicating promising preliminary results (see Figure S3). Besides, the performance quality of FNDs could also be boosted in considering the optimization aspects of monodispersity, NV-center density, and surface engineering. (ii) On the theory front, we can refine the mathematical model of the imaging system that quantifies the system interference, NV signal pattern, and noise statistics. (iii) On the algorithmic front, the data distortion like frame drift could possibly be solved by introducing an image registration method. Additionally, a comprehensive investigation into the point spread function (PSF) of our optical imaging system can unlock the potential for imaging with super-resolution capability beyond hardware limits.

Overall, the proposed approach features a multidimensional classifier with practical utility and effectiveness for noise-laden scenarios and is compatible with existing quantum sensing protocols. It is a valuable tool for advancing the capabilities and practicality of FND-based background-free quantum sensing and imaging in real-world applications, pushing the limits of current imaging techniques. This method also opens up new possibilities for promoting FNDs into broader biomedical applications like healthcare, tumor detection, and pathological tracking processes.

## Supporting Information Available

Recently concluded developments for background-removed imaging based on nanodiamonds; illustrations for the self-built optical wide-field setup, the lattice structure, and spectra of FNDs; widely tunable frequency range of FNDs (1–1000 Hz) by optically modulated polarization; Fourier fitting of NV and non-NV pixel data (frequency and amplitude) and the fitting accuracy map of all the pixel distributions; fluorescent spectrum of AF-Red dye and the detected ODMR spectra of FNDs; experimental protocols of selective addressing and enhanced ODMR detection of FNDs inside 3T3 cells; dynamic light scattering showing the size distribution of FND and its antibody-conjugates; selective immunofluorescent imaging of FNDs conjugated with SAb in brain slice; additional experimental methods details, materials, and methods; Fourier analysis of NV pixel signal; evaluation of the enhancement of signal-to-background ratio (PDF)

## References

[ref1] Yan Y.; Marriott G. Analysis of Protein Interactions Using Fluorescence Technologies. Curr. Opin. Chem. Biol. 2003, 7, 635–640. 10.1016/j.cbpa.2003.08.017. 14580569

[ref2] Ueno T.; Nagano T. Fluorescent probes for sensing and imaging. Nat. Methods. 2011, 8, 642–645. 10.1038/nmeth.1663. 21799499

[ref3] Liu X.; Chang Y. T. Fluorescent probe strategy for live cell distinction. Chem. Soc. Rev. 2022, 51, 1573–1591. 10.1039/D1CS00388G. 35136889

[ref4] Betzig E.; Patterson G. H.; Sougrat R.; Lindwasser O. W.; Olenych S.; Bonifacino J. S.; Davidson M. W.; Schwartz J. L.; Hess H. F. Imaging intracellular fluorescent proteins at nanometer resolution. Science 2006, 313, 1642–1645. 10.1126/science.1127344. 16902090

[ref5] Marriott G.; Mao S.; Sakata T.; Ran J.; Jackson D. K.; Petchprayoon C.; Gomez T. J.; Warp E.; Tulyathan O.; Aaron H. L.; Yan Y. Optical lock-in detection imaging microscopy for contrast-enhanced imaging in living cells. Proc. Natl. Acad. Sci. U. S. A. 2008, 105, 17789–17794. 10.1073/pnas.0808882105. 19004775 PMC2584753

[ref6] Dean K. M.; Palmer A. E. Advances in fluorescence labeling strategies for dynamic cellular imaging. Nat. Chem. Biol. 2014, 10, 512–523. 10.1038/nchembio.1556. 24937069 PMC4248787

[ref7] Li N.; Zhao R.; Sun Y.; Ye Z.; He K.; Fang X. Single-molecule imaging and tracking of molecular dynamics in living cells. Natl. Sci. Rev. 2017, 4, 739–760. 10.1093/nsr/nww055.

[ref8] Basu S.; Hendler-Neumark A.; Bisker G. Dynamic tracking of biological processes using near-infrared fluorescent single-walled carbon nanotubes. ACS Appl. Mater. Interfaces. 2024, 16, 54960–54975. 10.1021/acsami.4c10955. 39377262 PMC11492180

[ref9] Aubin J. E. Autofluorescence of viable cultured mammalian cells. J. Histochem. Cytochem. 1979, 27, 36–43. 10.1177/27.1.220325. 220325

[ref10] Wizenty J.; Ashraf M. I.; Rohwer N.; Stockmann M.; Weiss S.; Biebl M.; Pratschke J.; Aigner F.; Wuensch T. Autofluorescence: A potential pitfall in immunofluorescence-based inflammation grading. J. Immunol. Methods. 2018, 456, 28–37. 10.1016/j.jim.2018.02.007. 29458079

[ref11] Dean K. M.; Palmer A. E. Advances in fluorescence labeling strategies for dynamic cellular imaging. Nat. Chem. Biol. 2014, 10, 512–523. 10.1038/nchembio.1556. 24937069 PMC4248787

[ref12] Yao J.; Yang M.; Duan Y. Chemistry, biology, and medicine of fluorescent nanomaterials and related systems: new insights into biosensing, bioimaging, genomics, diagnostics, and therapy. Chem. Rev. 2014, 114, 6130–6178. 10.1021/cr200359p. 24779710

[ref13] Chang Y. R.; Lee H. Y.; Chen K.; Chang C. C.; Tsai D. S.; Fu C. C.; Lim T. S.; Tzeng Y. K.; Fang C. Y.; Han C. C.; Chang H. C.; Fann W. Mass production and dynamic imaging of fluorescent nanodiamonds. Nat. Nanotechnol. 2008, 3, 284–288. 10.1038/nnano.2008.99. 18654525

[ref14] Aharonovich I.; Greentree A. D.; Prawer S. Diamond photonics. Nat. Photonics. 2011, 5, 397–405. 10.1038/nphoton.2011.54.

[ref15] Tan Y.; Hu X.; Hou Y.; Chu Z. Emerging diamond quantum sensing in bio-membranes. Membranes 2022, 12, 957 10.3390/membranes12100957. 36295716 PMC9609316

[ref16] Fu C. C.; Lee H. Y.; Chen K.; Lim T. S.; Wu H. Y.; Lin P. K.; Wei P. K.; Tsao P. H.; Chang H. C.; Fann W. Characterization and application of single fluorescent nanodiamonds as cellular biomarkers. Proc. Natl. Acad. Sci. U. S. A. 2007, 104, 727–732. 10.1073/pnas.0605409104. 17213326 PMC1783382

[ref17] McGuinness L. P.; Yan Y.; Stacey A.; Simpson D. A.; Hall L. T.; Maclaurin D.; Prawer S.; Mulvaney P.; Wrachtrup J.; Caruso F.; Scholten R. E.; Hollenberg L. C. L. Quantum measurement and orientation tracking of fluorescent nanodiamonds inside living cells. Nat. Nanotechnol. 2011, 6, 358–363. 10.1038/nnano.2011.64. 21552253

[ref18] Jelezko F.; Wrachtrup J. Single defect centres in diamond: A review. Phys. Status. Solidi. A 2006, 203, 3207–3225. 10.1002/pssa.200671403.

[ref19] Igarashi R.; Sugi T.; Sotoma S.; Genjo T.; Kumiya Y.; Walinda E.; Ueno H.; Ikeda K.; Sumiya H.; Tochio H.; Yoshinari Y.; Harada Y.; Shirakawa M. Tracking the 3D rotational dynamics in nanoscopic biological systems. J. Am. Chem. Soc. 2020, 142, 7542–7554. 10.1021/jacs.0c01191. 32285668

[ref20] Kucsko G.; Maurer P. C.; Yao N. Y.; Kubo M.; Noh H. J.; Lo P. K.; Park H.; Lukin M. D. Nanometre-scale thermometry in a living cell. Nature 2013, 500, 54–58. 10.1038/nature12373. 23903748 PMC4221854

[ref21] Wilson E. R.; Parker L. M.; Orth A.; Nunn N.; Torelli M.; Shenderova O.; Gibson B. C.; Reineck P. The effect of particle size on nanodiamond fluorescence and colloidal properties in biological media. Nanotechnology 2019, 30, 385704 10.1088/1361-6528/ab283d. 31181558

[ref22] Yanagi T.; Kaminaga K.; Suzuki M.; Abe H.; Yamamoto H.; Ohshima T.; Kuwahata A.; Sekino M.; Imaoka T.; Kakinuma S.; Sugi T.; Kada W.; Hanaizumi O.; Igarashi R. All-optical wide-field selective imaging of fluorescent nanodiamonds in cells, in vivo and ex vivo. ACS Nano 2021, 15, 12869–12879. 10.1021/acsnano.0c07740. 34339180

[ref23] Jones Z. R.; Niemuth N. J.; Robinson M. E.; Shenderova O. A.; Klaper R. D.; Hamers R. J. Selective imaging of diamond nanoparticles within complex matrices using magnetically induced fluorescence contrast. Environ. Sci-Nano 2020, 7, 525–534. 10.1039/C9EN01008D.

[ref24] Robinson M. E.; Ng J. D.; Zhang H.; Buchman J. T.; Shenderova O. A.; Haynes C. L.; Ma Z.; Goldsmith R. H.; Hamers R. J. Optically detected magnetic resonance for selective imaging of diamond nanoparticles. Anal. Chem. 2018, 90, 769–776. 10.1021/acs.analchem.7b03157. 29131578 PMC11910996

[ref25] Singam S. K.; Motylewski J.; Monaco A.; Gjorgievska E.; Bourgeois E.; Nesládek M.; Giugliano M.; Goovaerts E. Contrast induced by a static magnetic field for improved detection in nanodiamond fluorescence microscopy. Phys. Rev. Appl. 2016, 6, 064013 10.1103/PhysRevApplied.6.064013.

[ref26] Doronina-Amitonova L. V.; Fedotov I. V.; Zheltikov A. M. Ultrahigh-contrast imaging by temporally modulated stimulated emission depletion. Opt. Lett. 2015, 40, 725–728. 10.1364/OL.40.000725. 25723417

[ref27] Hui Y. Y.; Su L. J.; Chen O. Y.; Chen Y. T.; Liu T. M.; Chang H. C. Wide-field imaging and flow cytometric analysis of cancer cells in blood by fluorescent nanodiamond labeling and time gating. Sci. Rep. 2014, 4, 5574 10.1038/srep05574. 24994610 PMC4081895

[ref28] Sarkar S. K.; Bumb A.; Wu X.; Sochacki K. A.; Kellman P.; Brechbiel M. W.; Neuman K. C. Wide-field in vivo background free imaging by selective magnetic modulation of nanodiamond fluorescence. Biomed. Opt. Express 2014, 5, 1190–1202. 10.1364/BOE.5.001190. 24761300 PMC3985990

[ref29] Chapman R.; Plakhoitnik T. Background-free imaging of luminescent nanodiamonds using external magnetic field for contrast enhancement. Opt. Lett. 2013, 38, 1847–1849. 10.1364/OL.38.001847. 23722764

[ref30] Igarashi R.; Yoshinari Y.; Yokota H.; Sugi T.; Sugihara F.; Ikeda K.; Sumiya H.; Tsuji S.; Mori I.; Tochio H.; Harada Y.; Shirakawa M. Real-time background-free selective imaging of fluorescent nanodiamonds in vivo. Nano Lett. 2012, 12, 5726–5732. 10.1021/nl302979d. 23066639

[ref31] Wang L.; Hou Y.; Zhang T.; Wei X.; Zhou Y.; Lei D.; Wei Q.; Lin Y.; Chu Z. All-optical modulation of single defects in nanodiamonds: revealing rotational and translational motions in cell traction force fields. Nano Lett. 2022, 22, 7714–7723. 10.1021/acs.nanolett.2c02232. 35946594

[ref32] Wang L.; Yu X.; Zhang T.; Hou Y.; Lei D.; Qi X.; Chu Z. High-dimensional anticounterfeiting nanodiamonds authenticated with deep metric learning. Nat. Commun. 2024, 15, 10602 10.1038/s41467-024-55014-2. 39638812 PMC11621400

[ref33] Alegre T. P. M.; Santori C.; Medeiros-Ribeiro G.; Beausoleil R. G. Polarization-selective excitation of nitrogen vacancy centers in diamond. Phys. Rev. B 2007, 76, 165205 10.1103/PhysRevB.76.165205.

[ref34] Zhu J.; Thompson C. B. Metabolic regulation of cell growth and proliferation. Nat. Rev. Mol. Cell Biol. 2019, 20, 436–450. 10.1038/s41580-019-0123-5. 30976106 PMC6592760

[ref35] Patton E. E.; Zon L. I.; Langenau D. M. Zebrafish disease models in drug discovery: from preclinical modelling to clinical trials. Nat. Rev. Drug Discovery 2021, 20, 611–628. 10.1038/s41573-021-00210-8. 34117457 PMC9210578

