# Supplementary material for: Selective Addressing of Versatile Nanodiamonds via Physically-Enabled Classifier in Complex Biosystems
Source: Nano Lett. 2025 Mar 14;25(14):5679–87. doi: 10.1021/acs.nanolett.4c06567 (PMC11987062; doi:10.1021/acs.nanolett.4c06567)
Supplement: Supplementary file 1 — nl4c06567_si_001.pdf [file nl4c06567_si_001.pdf]

Supporting Information

Selective Addressing of Versatile Nanodiamonds via Physically-Enabled Classifier in Complex Bio-Systems

Yayin Tan[1a], Xiaolu Wang[2a], Feng Xu[1], Xinhao Hu[1], Yuan Lin[1], Bo Gao[2, 3] and Zhiqin Chu[1]**

[1]Department of Electrical and Electronic Engineering, the University of Hong Kong, Pok Fu Lam, Hong Kong, China.

[2]School of Biomedical Sciences, Faculty of Medicine, the Chinese University of Hong Kong, Shatin, Hong Kong, China

[3]Centre for Translational Stem Cell Biology, Tai Po, Hong Kong, China.

Email: bogao@cuhk.edu.hk (Bo Gao); zqchu@eee.hku.hk (Zhiqin Chu)

Table S1. The recent developments for background removed imaging based on nanodiamonds.

Method	ND Contrast ($\frac{\Delta I}{I_{max}}$)	Signal-to-background Ratio (SBR in dB)	Modulation Method	Signal Acquisition	Modulation Speed	Year
Our method: Selective addressing of nanodiamonds via physically-enabled classifier with optically modulated polarization	*20% - 68%*	*Improved from 2.2 to 68.7*	*Optically modulated by HWP*	*Fluorescent signal in image form*	*~ 30 ms for 1-degree modulation*	*2024*
Optical NDs selective imaging of FNDs by pulse sequences [22]	3.3%-4.2%	SBR: improved from 1.31 to 20.7 in live C. elegans nematode; from 3.9 to 18.7 on an ex vivo hippocampal slice; from 3.2 to 43.9 and from 4.01 to 71.08 of different bright spots in a live cell.	Laser pulse sequences: controlled by changing the pulse recurrence intervals of microsecond excitation laser pulses	Fluorescent image	Frame exposure time: 33/100 ms<br>Pulse width: 5 µs<br>Long intervals: 100 µs<br>Short intervals: 2 µs	2021
Selective Imaging of Diamond Nanoparticles using Magnetically Induced Fluorescence Contrast [23]	6% - 10% in practical imaging	S/N: >13	Magnetic fields	Fluorescent image	Magnet setting: 4 points with magnet off, 4 points with magnet on (8 s)	2020

ODMR for Selective Imaging of Diamond Nanoparticles [24]	ODMR contrast: 0.4% - 3%	Not mentioned	Resonant microwave field	ODMR image and spectra	Microwave: amplitude-modulated at 200 Hz with on-resonance (2.87 GHz) and then off-resonance (2.80 GHz)	2018
Contrast Induced by a Static Magnetic Field for Improved Detection in Nanodiamond Fluorescence Microscopy [25]	Intensity-dependent Contrast (FND): Low excitation rates: 6%-13%) High excitation rates: 20%-38%	*"Limited SNR"*	Static magnetic field and microwave	Fluorescent image	Not mentioned	2016
Optical ultrahigh-contrast imaging by temporally modulated stimulated emission depletion [26]	Not mentioned	Image contrast: $\eta = exp\ (\sigma\ I_{STED}) \approx 120$	Temporally modulated STED with lock-in detection	Fluorescent image	STED modulation frequency: 415 Hz	2015
Wide-field imaging by FND time gating [27]	Not mentioned	Image contrast: ~20	Fluorescence time gating	Fluorescent image	Imaging system: a frame rate of 23 Hz in 60 s<br>The gating time of the ICCD: 10 ns	2014

Wide-field background free imaging by selective magnetic modulation [28]	~6%	SBR: improved from ~12 to ~50	Magnetic modulation for isolated FND MW modulation demonstrated in vivo imaging	Fluorescent image	Acquisition time of 250 ms per image	2014
Background-free imaging of NDs using external magnetic field [29]	Not mentioned	Not mentioned	External magnetic field (25 mT)	Fluorescent image	Not mentioned	2013
Background-Free Selective Imaging of FNDs in Vivo [30]	2.8% - 5.6%	Not mentioned	Spin state modulated by MW irradiation	Fluorescent image & ODMR spectrum	MW irradiation on and off every 10 ms	2012

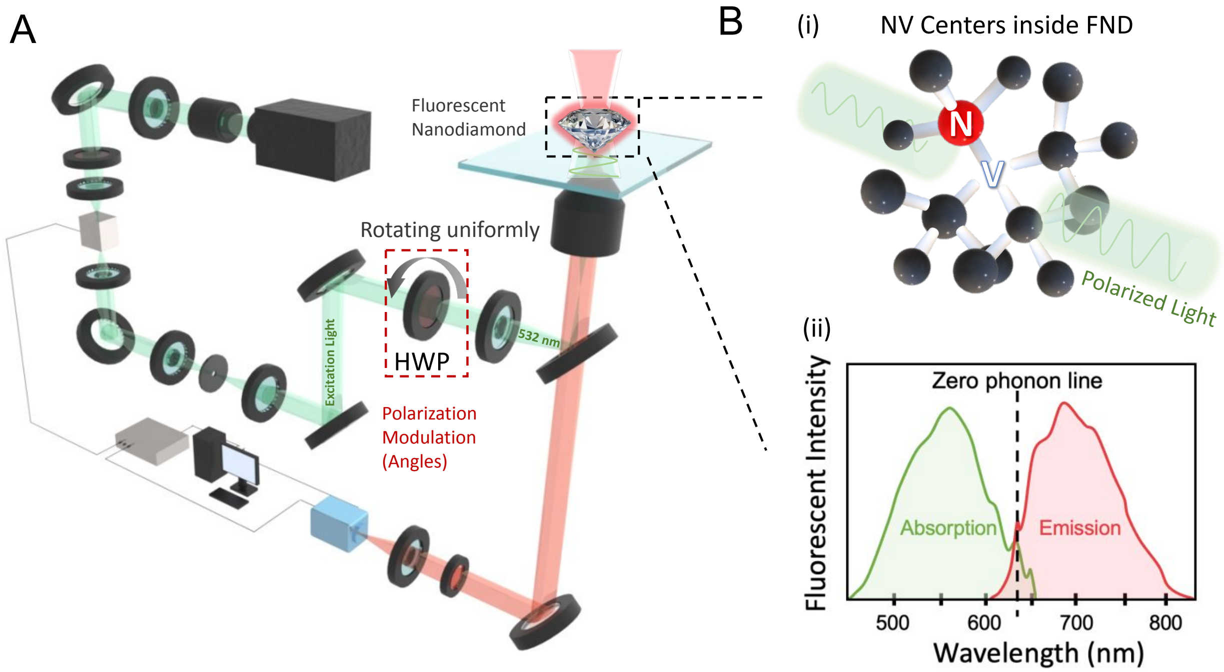

Figure S2. Illustrations for the self-built optical wide-field setup, the lattice structure and spectra of FNDs. A) The self-built optical wide-field setup for the imaging. B) (i) The lattice structure of FNDs containing NV centers inside. (ii) The absorption and emission spectra of NV fluorescence.

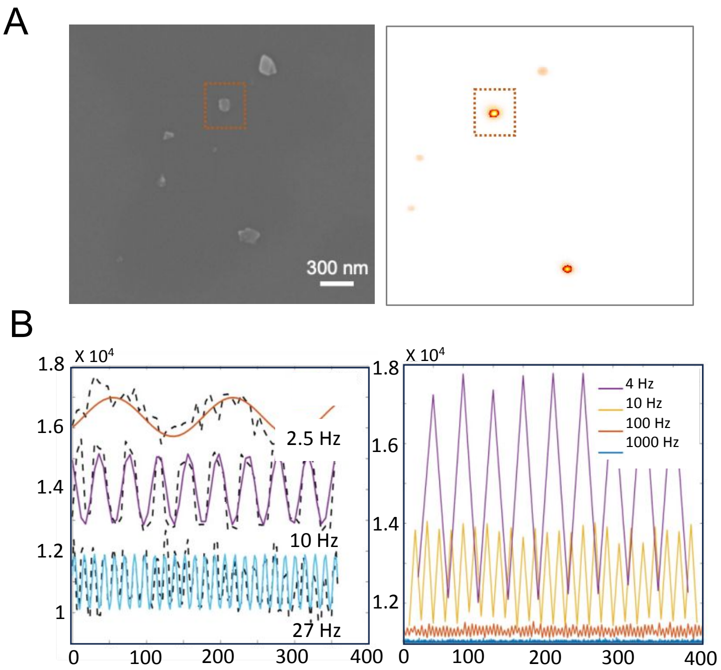

Figure S3. The widely-tunable frequency range of FNDs (1 - 1000 Hz) by optically modulated polarization. A) Left: the SEM image of FNDs. Right: the wide-field fluorescent image of FNDs, without background disturbances. B) Left: tunable frequency range by EMCCD. Right: tunable frequency range by event camera.

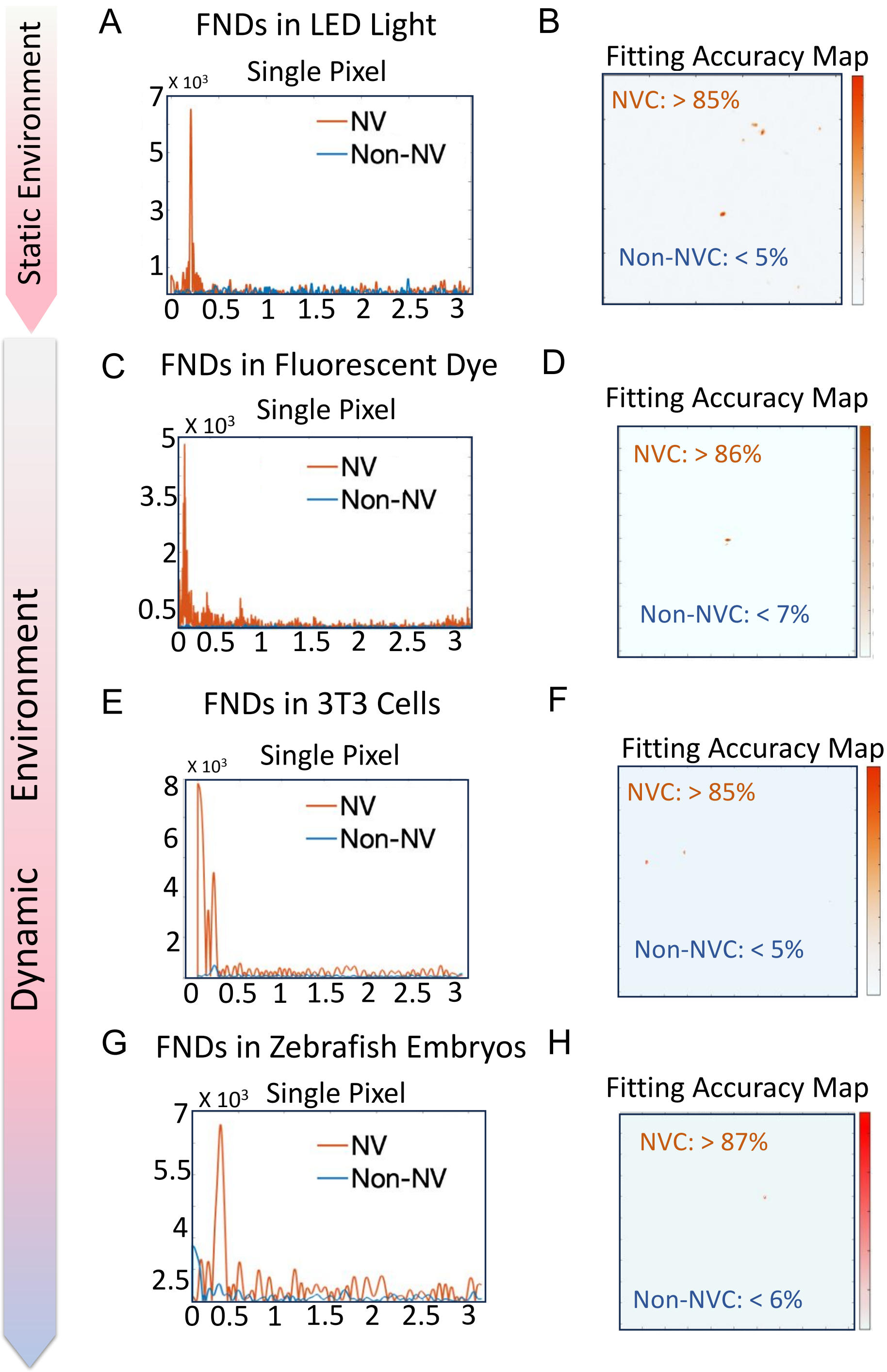

Figure S4. The Fourier fitting of NV and non-NV pixel data (frequency and amplitude) and the fitting accuracy map of all the pixel distributions. A, C, E, G) The Fourier fitting of NV and non-NV pixel signals shown in distinct comparison. B, D, F, H) The fitting accuracy map of all the pixel distributions, providing multi-dimensional features for selective imaging of FNDs. The

fitting accuracy is employed as the power ratio between the fitted sinusoid and original signal, an evaluation metric to describe the model match of the measurements. More concretely, a value of fitting accuracy close to 100% indicates a perfect sinusoidal fitting; while a value close to 0% shows a complete mismatch between the data and model.

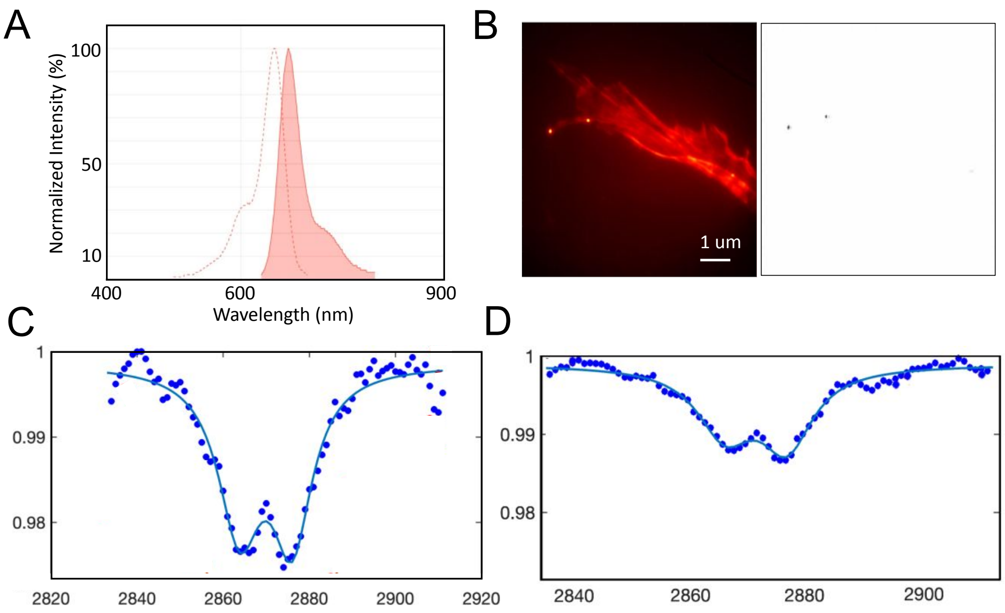

Figure S5. Fluorescent spectrum of AF-Red dye and the detected ODMR spectra to confirm selective imaged FNDs as bright spots. A) Fluorescent spectrum of AF-Red dye. B) Fluorescent image of FNDs inside AF-Red stained cell and its selective imaging of FNDs. C,D) The ODMR spectra of the FNDs in (B), with 91% and 90% confidence interval of the Lorentz fit for (C) and (D), respectively.

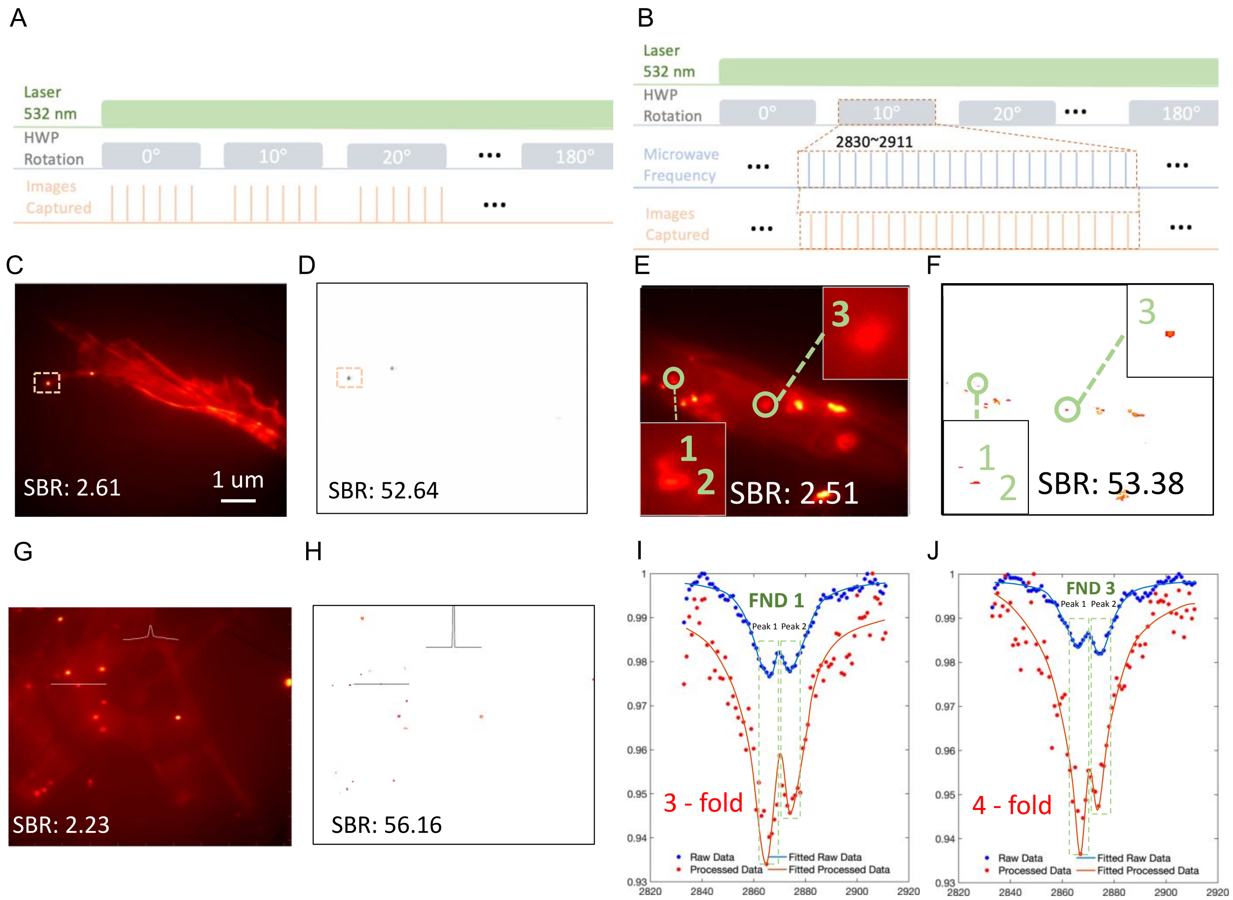

Figure S6. The experimental protocols of selective addressing and enhanced ODMR detection of FNDs inside 3T3 cells. A) Illustration for the experimental protocol of selective addressing of FNDs inside cells. B) Illustration for the experimental protocol of ODMR measurements of FNDs inside cells. C, E, G) The fluorescent images of FNDs inside cells captured by self-built wide-field microscope. D, F, H) The selective fluorescent imaging of FNDs inside cells by the classifier, where the background fluorescence of the stained cell is removed. I, J) The ODMR curves of FND 1 and FND 3 in green circles of (E) with 3 and 4-fold improved contrasts via the classifier. The fitting results are represented with 90% confidence interval of the Lorentz fit.

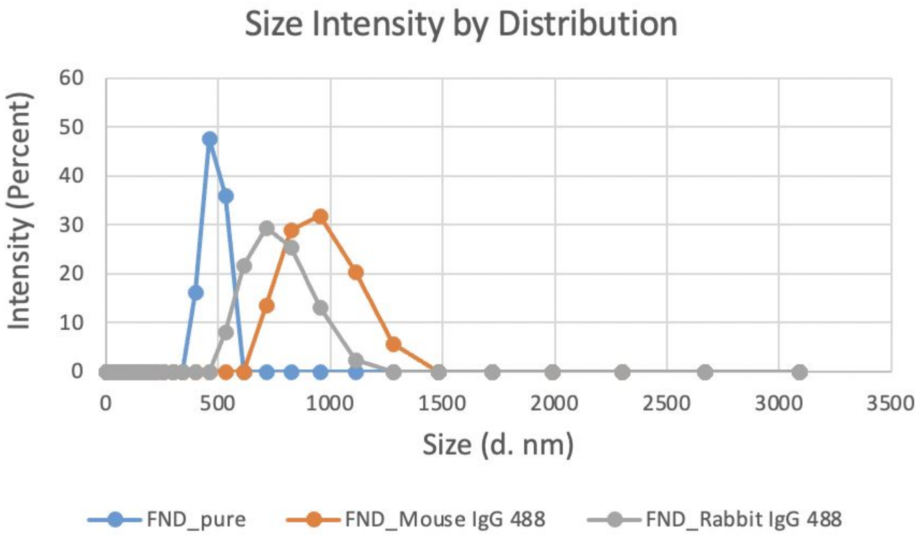

Figure S7. The dynamic light scattering (DLS) showing the size distribution of FND and its antibody-conjugates in PBS.

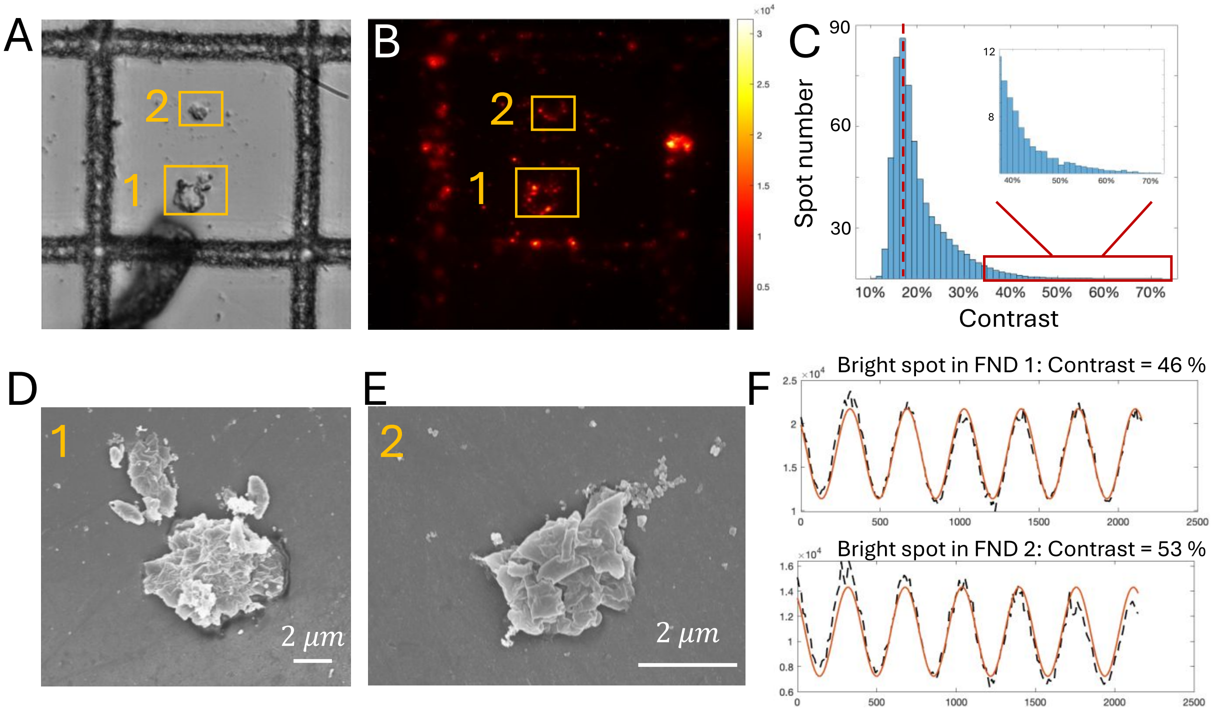

Figure S8. Characteristics of aggregated FNDs containing large NV concentrations under specifically-built wide-field system. (A) Wide-field optical image of FNDs. (B) Wide-field fluorescent image of FNDs. (C) Histogram of FND contrast distribution among all the identified

bright pixel spots in (B). (D) SEM image of aggregated FND 1 shown in (A) and (B). (E) SEM image of aggregated FND 2 shown in (A) and (B). The original size of FNDs is 100 nm. (F) NV signal curves of bright spots in FND 1 and FND 2 corresponding to the rectangle areas shown in (A).

EXPERIMENTAL METHODS

Preparations of the detected FNDs. The FNDs used were carboxylated FNDs hosting ensemble NV centers (BR100, FND Biotech, Inc.), which were attached to cover slides through electrostatic adsorption. Initially, cover slides were polished and activated using plasma for 10 minutes at 200 W. They were then immersed in a 5% solution of 3-aminopropyltriethoxysilane (APTES, Sigma) in ethanol for 24 hours reaction at room temperature. Following this, the cover slides were cleaned with ethanol and water. Subsequently, 0.02 mg/mL solution of FNDs was drop-casted onto the positively charged cover glass and kept in the refrigerator to incubate for 3 hours. After incubation, the samples were rinsed with DI water and air-dried. Finally, a PDMS film was applied to the cover glass for protection. The size of FND conjugates was characterized by dynamic light scattering (DLS) (Figure S7). The pure FND hydrodynamic size ranged between 342 and 521 nm. As expected, the size of IgG 488 @ FND increases after the antibody conjugation. It can be observed that the FND size changed from ~350 nm (Pure FNDs) to ~750 nm (FND conjugations) as evidence of IgG 488 being successfully conjugated with FNDs.

Optical modulation using rotating HWP in Figure 1 – the relations with β (laser polarization angle), α (NV projection angle), and θ (modulation contrast angle) The HWP can be controlled for constant rotation to change the polarization direction of linearly polarized excitation laser uniformly, where the polarized angle of excitation laser (green arrow) is defined as β. As the HWP rotates, the NV fluorescent intensities with uniformly-varying β values are collected frame-by-frame as a series of fluorescent images by using an EMCCD camera, and its intensity at each image pixel can be extracted to form one-dimensional curves changing with the polarization angle β. The inserts present the relationship between the NV projection direction onto the sample plane and the laser polarization direction, corresponding to I_{max} (β) (yellow dot)

and I_{min} (β) (red dot), respectively. (|α − β| = 0° or 180°). The high contrast value of modulated FNDs is determined by θ. Specifically, as indicated by equation (1), a larger θ results in a higher contrast value, leading to improved performance of the modulation.

Construction of the optically modulated imaging system. All fluorescent images for the selective addressing of FNDs were captured using a custom-built wide-field fluorescence microscope. A continuous 532 nm laser was used in the optical path for excitation, whose linear polarization was adjusted using a half-wave plate (WPH10M-532, Thorlabs) mounted on an electrical rotation stage (PT-GD62, PDV). The HWP can be controlled for constant rotation to change the polarization direction of linearly polarized excitation laser uniformly, where the polarized angle of excitation laser (green arrow) is defined as β in Figure 1A(i-3). As the HWP rotates, the NV fluorescent intensities with uniformly-varying β values are collected frame-by-frame as a series of fluorescent images by using an EMCCD camera, and its intensity at each image pixel can be extracted to form one-dimensional curves changing with the polarization angle β, illustrated in Figure 1A. When the NV axis forms a 90° degree with the excitation laser ($|\alpha - \beta| = 90°$) on the sample plane, NV signal reached to its maximum intensity. When the NV axis coincides with the excitation laser direction on the sample plane, NV signal reduced to its minimum intensity as shown in Figure 1A. The rotation speed of the half-wave plate can be flexibly adjusted to change the fluorescent signal frequency. This excitation laser beam was focused through the back-focal plane of an oil immersion objective (NA 1.45, UPLXAPO100XO, Olympus) to effectively illuminate the sample. The position of the sample can be finely controlled using a nano-positioning stage (P561.3CD, Physik Instrumente). For detection optical path, the fluorescence signals were filtered through a long pass filter (FELH0650, Thorlabs), and captured by a water-cooled EMCCD (iXon Ultra 897, Andor). The

field of view of 40× object was around 75 μm × 75 μm and of the oil 100× object was around 30 μm × 30 μm.

Data Acquisition in Wide-Field Imaging System. The fluorescent images were captured using a self-built wide-field microscope with the excitation laser's linear polarization direction adjusted by 6 degrees per step, an integration time of 50 ms, and a laser power of about 10 mW. The half-wave plate in the microscope was set to rotate continuously, taking 3.25 seconds for each 180-degree rotation circle. During this rotation of the half-wave plate, the EMCCD camera captured images every 30 ms to record the signal changes of NV centers at varied excitation laser polarization angles, producing a sequence of images. For an efficient data representation, we algebraically encoded those 2D images of interest obtained at equidistant excitation polarization angles as a 3D tensor (tensor size: $H \times W \times K$. H, W: 2D image size, K: number of polarization angles), serving as the inputs to the designed imaging framework. The image resolution was 512 × 512. Each pixel at the same position across images were extracted as a one-dimensional signal, a sequence varying with the polarization angles of excitation laser. The corresponding pixel signal of each image was then denoised, fitted and analyzed *via* the self-designed Fourier fitting algorithm in MATLAB. The frequency, amplitude, and fitting accuracy of each pixel signal were then assessed using the physically-enabled classifier designed to leverage physical properties. This process significantly enhanced the precision and effectiveness of selectively addressing NV signals based on these features. SBR values for each image were calculated and digitized. For ODMR measurements, during the continuous-time laser irradiation, the HWP rotated uniformly from 0° to 180° with 10° step size (See Figure S6B). For each polarization angle, the microwave frequency was tuned to sweep in a certain range from 2830 MHz to 2911 MHz, where the 2D images are captured under each microwave frequency.

Incubation of FNDs into the interior of 3T3 cells. 3T3 cells were obtained from ATCC (CAT#CRL-1658) and were cultured in Dulbecco's modified Eagle's medium (DMEM, ThermoFisher, # 12491015), supplemented with 10% Fetal Bovine Serum (FBS, ThermoFisher, #A5669701) and 1% Penicillin-Streptomycin (ThermoFisher, #15140122). Cells were seeded onto slides in 24 well plates and grown to 50% confluence. After 24 hours incubation, the culture medium was removed and replaced with fresh medium containing 100 μg/mL FND. Cells were rinsed three times with PBS after 24 hours culture and fixed with 4% paraformaldehyde (PFA, Sigma Aldrich, #158127, U.S.) in PBS at room temperature for 10 minutes. 0.5% Triton X-100 was used to treat fixed cells for 5 mins to enhance the permeability of cell membrane. After being washed for 3 times with PBS, cells were stained with Alexa Fluor™ 647 Phalloidin (ThermoFisher, #A22287) for 1 hour at 4°C protected from light. Again, with three washes with 0.1% Triton X-100/PBS, the slides were mounted with ProLong Diamond Antifade Mountant (ThermoFisher, #P36961) and were subjected for confocal imaging and classifier analysis.

The microinjection of FND particles into Zebrafish embryos. Zygotes obtained from wild type (TU) mating pairs were used for FND injection. FND particles were suspended in double-distilled water (ddH_2O) with a concentration of 250 ng/μL. The microinjection was performed using a Nikon SMZ 745T stereomicroscope equipped with a Warner Picoliter Injector PLI-90A and a 3D manual micro-manipulator fitted with a micropipette having an outer diameter of 1 mm. A 1 nL drop of FND in ddH_2O was injected into the zygotes at the one-cell stage. At 24 hours post fertilization (hpf), all zebrafish embryos were imaged and ready for use.

The fabrication process of functional FND conjugations for immunofluorescent labelling. The FND conjugations were fabricated under strategy of modifying the FND surface with functional groups and then conjugating functional FNDs with the secondary antibody Goat anti-

Rabbit IgG (H+L) Cross-Adsorbed Secondary Antibody, Alexa Fluor™ 488 (SAb, Thermofisher Cat No. A-11008). Firstly, 1 mg of acid-treated FND (FND-COOH) was dispersed in acidic ddH_2O (pH ≈ 4) *via* sonication for 15 minutes. Then, 2 mg of N-(3-dimethylaminopropyl)-N′-ethylcarbodiimide hydrochloride (EDC, Sigma-Aldrich, Cat No. 22980) and 2 mg of N-hydroxysuccinimide (NHS, Sigma-Aldrich, Cat No. 24500) were added to the FND dispersion for a 30-minute reaction, activating the surface carboxyl groups of FNDs. Later, the activated FNDs were separated by the centrifugation at 20,000 × g for 10 minutes and were washed with basic ddH_2O (pH ≈ 8). The activated FND particles were then mixed with 5 mg O-(2-aminoethyl)-O'-(2-carboxyethyl) polyethylene hydrochloride (NH2-PEG-COOH, molecular weight ≈ 3000, Sigma-Aldrich) to react for 6 hours. This step PEGylates the FND particles. Later on, the PEGylated FND particles were thoroughly washed with neutral ddH_2O. The carboxyl groups on the PEGylated FND particles were activated again by adding EDC and NHS (similar to step 2). Soon afterwards, Goat Anti-Rabbit IgG 488 (200 μg) was added to the activated PEGylated FND particles and allowed to react for 5 hours to attach Goat Anti-Rabbit IgG 488 onto the functional surface of FND particles. The Goat Anti-Rabbit IgG 488 @FND conjugations were then coated with Bovine Serum Albumin (BSA) by mixing them with 1 mg of BSA for them to react for 2 hours. This step blocks any residual open sites on the particle surfaces. Next, the Goat Anti-Rabbit IgG 488 @FND conjugates were separated from the solution by centrifugation at 20,000 × g for 10 minutes. The separated conjugates were then washed three times with phosphate-buffered saline (PBS) and dispersed in a 0.5% BSA/PBS solution for longer time storage at 4 °C.

Immunofluorescence staining of mouse brain slice with FND conjugates. Cryosections of adult mouse brain (postnatal 60 days) were washed three times in 1% Triton-X/PBS for 5

minutes each. They were then blocked in 10% goat serum (Sigma Aldrich, #G9023, U.S.) in 1% Triton-X/PBS for 1-2 hours at room temperature. Next, the sections were incubated with a primary antibody diluted in blocking solution overnight at 4 °C with rotation (Anti-NeuN antibody, ab177487, Abcam). Next day, the brain slices were washed five times in 1% Triton-X/PBS for 10 minutes each at room temperature. Subsequently, the slices were incubated with a secondary antibody diluted in blocking solution (Goat anti-Rabbit IgG (H+L) Alexa Fluor 488 conjugated with FND) for 2 hours at room temperature (or overnight at 4 °C). After three washes with 1% Triton-X/PBS, the slices were mounted with ProLong Diamond Antifade Mountant with DAPI (ThermoFisher Scientific, #P36962, U.S.) and examined using Zeiss LSM800 confocal microscopy and wide-field microscopy. The acquired images were analyzed using Zeiss Zen software. Control group 1 was to utilize the control IgG 488 as the immunofluorescent labelling SAb without FNDs inside the neuronal slice, seen in the second row of Figure 4B. Here, we found that the control IgG 488 signals were dominantly localized in the nucleus regions (co-localization with DAPI) in brain slices. In the negative control group 2, mouse brain slices were incubated with FND solution, seen in the third row of Figure 4B. Here, mouse brain slices were also stained with DAPI. We were unable to detect any FND signals in 488 nm and 647 nm channel, since FNDs without specific conjugations won't be specifically labeled in mouse brain slices. This authenticates the specific binding function of FND conjugations in a reverse verification way.

Study approval. Zebrafish experiments were conducted in compliance with the guidelines of the Committee on the Use of Laboratory Animals for Teaching and Research (CULATR) at the University of Hong Kong (Approval No. CULATR 5396-20). Mouse experiments were conducted in compliance with CULATR 5447-20 at the University of Hong Kong.

Fourier analysis of NV pixel signal

Consider a K-point one-dimensional NV signal x[k], sampled from a three-dimensional tensor at pixel coordinate (i, j) of an image as shown in Fig. 1B:

$$x[k] = \left(\frac{8}{9}\, I_{actual}\sin^2(\theta)\right)cos(\omega_0 k + \varphi) + \frac{1}{9}\, I_{actual}, k \ = (1)$$

Where I_{actual} is the actual excitation laser power, K is the number of experimental polarization angles (see Fig. 1B in the main text), and φ is the initial phase offset. As introduced in the main text (Fig. 1A), the parameter θ is constant of NV axis, while ω_0 is the rotation angular frequency. This connects the equation (1) above to equation (1) in the main text.

Based on the imaging framework, each NV signal is characterized by a single sinusoid, of which its frequency is determined by the tunable rotation speed of the HWP, i.e. ω_0. The main goal here is to estimate ω_0 and its associated amplitude a_0 ($a_0 = \frac{8}{9} I_{actual}\sin^2(\theta)$) from measurements x[k]. One simple approach is to use the Discrete Fourier Transform (DFT) of x[k] :

$$X[m] = \sum_{k=0}^{K-1} x[k]\, e^{\frac{-2j\pi km}{K}} ,m \ = \ 0,1,\ldots,K-1 (2)$$

This method is straightforward, simple and efficient. However, it suffers from limited resolution, i.e., $\frac{2\pi}{K}$, where K is the number of experimental polarization angles. This leads to a poor resolution in practice, particularly when the acquisition time is limited. Hence, we need to leverage another solution with higher resolution beyond DFT.

Here, we utilized advanced Fourier fitting method based on annihilation filter approach. To estimate *N* frequencies ($\omega_1,\omega_2,...\omega_N$) from a sequence of samples x[k],

$$x[k] = \sum_{n=1}^{N} a_n\, e^{j\omega_n k} \quad , k = 0, 1, ..., K-1.$$

We constructed a time-domain filter for which its *z*-transform is defined as,

$$H(z) = \sum_{n=0}^{N} h[n] z^{-n} = \prod_{n=1}^{N} (1 - e^{j\omega_n} z^{-1}) (3)$$

Then, we demonstrated that h[k] "annihilates" the x[k] as follows:

$$h_k * x_k = \sum_{n=0}^{N} h_n x_{k-n} = \sum_{n=0}^{N}\sum_{m=1}^{N} h_n a_m\, e^{j\omega_m (k-n)} = \sum_{m=1}^{N} a_m e^{j\omega_m k} \underbrace{\sum_{n=0}^{N} h_n e^{-j\omega_m n}}_{= H(e^{j\omega_m}) = 0} = 0 \quad (4)$$

The equation (4) offers a system of linear equations, resulting in the coefficient h[n]. It implies that filter h annihilates the sinusoidal samples. The equation (4) follows from the fact that ($e^{j\omega_n}$, n = 1,...,N) is the root of the polynomial H(z). While the linear parameters a_n can be easily recovered via least-squares, which again is a linear problem. In the presence of background noise, the single-frequency estimation for amplitude a_0 can be formulated as:

$$a_0 = \arg \min_{a_0 \in \mathbb{C}} \sum_{n=1}^{N-1} |x[n] - a_0 e^{jn\omega_0}|^2$$

In summary, we estimated ω_0 using Prony-based method and find the amplitude a_0 using the least-squares fit.

1. Frequency: estimate ω_0 using Prony's method described above

2. Amplitude: estimate a_0 using Least-Squares

$$a_0 = \arg \min_{a_0 \in \mathbb{C}} \sum_{n=1}^{N-1} |x[n] - a_0 e^{jn\omega_0}|^2$$

The recovered NV signal contaminated by noise was processed by the frequency estimation algorithm. The ground-truth frequency obtained from experimental setup is 0.2238, while the estimated frequency is 0.2191. The imaging quality is further evaluated by SBR (signal-to-background ratio), defined by

$$\mathrm{SBR} = 10\ \log_{10}\left(\frac{\mathrm{MAX}_x^2}{\mathrm{MSE}}\right)$$

$$\mathrm{MSE} = \frac{1}{N}\sum_{n=0}^{N-1}|\tilde{x}_n - x_n|^2$$

($\tilde{x}_n$-reconstruction, x_n- ground-truth)

Where $\mathrm{MAX}_x = \max_i |x_i|$ (maximum pixel intensity) and MSE is the mean squared error between the ground-truth and the reconstruction.

REFERENCES

22. Yanagi, T.; Kaminaga, K.; Suzuki, M.; Abe, H.; Yamamoto, H.; Ohshima, T.; Kuwahata, A.; Sekino, M.; Imaoka, T.; Kakinuma, S.; Sugi, T.; Kada, W.; Hanaizumi, O.; Igarashi, R. All-optical wide-field selective imaging of fluorescent nanodiamonds in cells, in vivo and ex vivo. *ACS nano* **2021,** 15, 12869-12879.

23. Jones, Z. R.; Niemuth, N. J.; Robinson, M. E.; Shenderova, O. A.; Klaper, R. D.; Hamers, R. J. Selective imaging of diamond nanoparticles within complex matrices using magnetically induced fluorescence contrast. *Environ Sci-Nano* **2020,** 7, 525-534.

24. Robinson, M. E.; Ng, J. D.; Zhang, H.; Buchman, J. T.; Shenderova, O. A.; Haynes, C. L.; Z. Ma, Z.; Goldsmith, R. H.; Hamers, R. J. Optically detected magnetic resonance for selective imagingof diamond nanoparticles. *Anal. Chem.* **2018,** 90, 769-776.

25. Singam, S. K.; Motylewski, J.; Monaco, A.; Gjorgievska, E.; Bourgeois, E.; Nesládek, M.; Giugliano, M.; Goovaerts, E. Contrast induced by a static magnetic field for improved detection in nanodiamond fluorescence microscopy. *Phys. Rev. Appl.* **2016,** 6, 064013.

26. Doronina-Amitonova, L. V.; Fedotov, I. V.; Zheltikov, A. M. Ultrahigh-contrast imaging by temporally modulated stimulated emission depletion. *Opt. Lett.* **2015,** 40, 725-728.

27. Hui, Y. Y.; Su, L. J.; Chen, O. Y.; Chen, Y. T.; Liu, T. M.; Chang, H. C. Wide-field imaging and flow cytometric analysis of cancer cells in blood by fluorescent nanodiamond labeling and time gating. *Sci. Rep.* **2014,** 4, 1-7.

28. Sarkar, S. K.; Bumb, A.; Wu, X.; Sochacki, K. A.; Kellman, P.; Brechbiel, M. W.; Neuman, K. C. Wide-field in vivo background free imaging by selective magnetic modulation of nanodiamond fluorescence. *Biomed. Opt. Express* **2014,** 5, 1190-1202.

29. Chapman, R.; Plakhoitnik, T. Background-free imaging of luminescent nanodiamonds using external magnetic field for contrast enhancement. *Opt. Lett.* **2013,** 38, 1847-1849.

30. Igarashi, R.; Yoshinari, Y.; Yokota, H.; Sugi, T.; Sugihara, F.; Ikeda, K.; Sumiya, H.; Tsuji, S.; Mori, I.; Tochio, H.; Harada, Y.; Shirakawa, M. Real-time background-free selective imaging of fluorescent nanodiamonds in vivo. *Nano Lett.* **2012,** 12, 5726-5732.
